# *Bacteroides acidifaciens* in the gut plays a protective role against CD95-mediated liver injury

**DOI:** 10.1080/19490976.2022.2027853

**Published:** 2022-02-06

**Authors:** Hesuiyuan Wang, Qing Wang, Chengmao Yang, Mingming Guo, Xiaoyue Cui, Zhe Jing, Yujie Liu, Wanjin Qiao, Hang Qi, Hongyang Zhang, Xu Zhang, Na Zhao, Mengjuan Zhang, Min Chen, Song Zhang, Haijin Xu, Liqing Zhao, Mingqiang Qiao, Zhenzhou Wu

**Affiliations:** a College of Life Sciences, Nankai University, Tianjin, China; b Department of General Surgery, Tianjin Medical University General Hospital, Tianjin, China; c The Key Laboratory of Molecular Microbiology and Technology, Ministry of Education, College of Life Sciences, Nankai University, Tianjin, China

**Keywords:** Hepatitis, gut microbiota, intestinal flora dysbiosis, CD95, GSH

## Abstract

The intestinal flora plays an important role in the development of many human and animal diseases. Microbiome association studies revealed the potential regulatory function of intestinal bacteria in many liver diseases, such as autoimmune hepatitis, viral hepatitis and alcoholic hepatitis. However, the key intestinal bacterial strains that affect pathological liver injury and the underlying functional mechanisms remain unclear. We found that the gut microbiota from gentamycin (Gen)-treated mice significantly alleviated concanavalin A (ConA)-induced liver injury compared to vancomycin (Van)-treated mice by inhibiting CD95 expression on the surface of hepatocytes and reducing CD95/CD95L-mediated hepatocyte apoptosis. Through the combination of microbiota sequencing and correlation analysis, we isolated 5 strains with the highest relative abundance, *Bacteroides acidifaciens* (BA), *Parabacteroides distasonis* (PD), *Bacteroides thetaiotaomicron* (BT), *Bacteroides dorei* (BD) and *Bacteroides uniformis* (BU), from the feces of Gen-treated mice. Only BA played a protective role against ConA-induced liver injury. Further studies demonstrated that BA-reconstituted mice had reduced CD95/CD95L signaling, which was required for the decrease in the L-glutathione/glutathione (GSSG/GSH) ratio observed in the liver. BA-reconstituted mice were also more resistant to alcoholic liver injury. Our work showed that a specific murine intestinal bacterial strain, BA, ameliorated liver injury by reducing hepatocyte apoptosis in a CD95-dependent manner. Determination of the function of BA may provide an opportunity for its future use as a treatment for liver disease.

## Introduction

Liver diseases, such as autoimmune hepatitis, viral hepatitis, alcoholic liver injury, and drug-induced liver injury, have attracted increasing attention due to their high morbidity and mortality rates.^1^ Mouse models that simulate human liver diseases aid investigations of the pathogenesis and therapies for liver diseases. Concanavalin A (ConA)-induced hepatitis is a well-established T cell-mediated and interferon-γ (IFN-γ)-dependent mouse model that simulates human autoimmune hepatitis.^2–4^ Alcohol and acetaminophen (APAP)-induced mouse liver injury models were used to study therapeutic options for human alcohol and drug-induced liver injury.^5^ The CD95/CD95L signaling pathway plays an important role in cell apoptosis. Activation of CD95/CD95L signaling directly induces liver damage in mice.^6^ CD95/CD95L-dependent hepatocyte apoptosis is widely involved in a variety of liver diseases,^7,8^ such as alcohol-induced liver injury,^9^ viral hepatitis,^10,11^ and hepatocellular carcinoma.^12^ Various factors, such as pathogen infection, cell death, antigen stimulation, cytokine stimulation, living conditions, and variations in the intestinal flora, greatly affect CD95 liver expression.^13–16^

Extensive research on the “intestinal flora-liver interaction” showed that intestinal bacteria were associated with many liver diseases, such as autoimmune hepatitis,^17^ alcoholic hepatitis,^18^ nonalcoholic hepatitis^19^ and viral hepatitis.^20^ A fecal microbiome analysis revealed that enrichment of *Veillonella dispar* in human intestines was related to the severity of autoimmune hepatitis.^21^ Fecal microbiota transplantation prevents alcohol-induced liver injury in alcohol-sensitive mice.^22,23^ The gut microbiota in mice may modulate CD95/CD95L signaling and alter the sensitivity to liver injury upon ConA challenge.^14^ An inhibition of CD95 expression was also observed with decreased hepatocellular apoptosis and alleviated liver injury in mice with autoimmune hepatitis administered *Akkermansia muciniphila*.^24^ Regulation of hepatocellular CD95 expression by the intestinal flora is closely associated with liver health and sensitivity to liver diseases.

*Bacteroides acidifaciens* (BA), which belongs to the *Bacteroidetes* phylum, is characterized as one of the predominant species in intestinal commensal bacteria.^25^ The relative abundance of BA in the gut was enhanced by a high soluble fiber diet.^26,27^ BA prevents obesity and increases insulin sensitivity.^28^ The role of BA in liver disease has rarely been studied. A previous study showed that BA expansion led to decrease of hepatic nonestesterified fatty acid level within high-fat diet mice.^29^ BA also contributes to acetate production to protect against nonalcoholic steatohepatitis (NASH) development.^30^ However, the regulatory function of BA in the “gut-liver axis” remains unclear.

The metabolites produced by intestinal bacteria and their derivatives enter the liver through the portal vein circulation and have regulatory functions in various cell types in the liver. Primary-to-secondary bile acid conversion mediated by intestinal Gram-positive bacteria enhances hepatic CXCR6^+^ nature kill T cell accumulation and inhibits liver tumor growth.^31^ The fecal levels of short-chain fatty acids (SCFAs) produced by the gut microbiota are decreased in patients with alcoholic hepatitis.^32^ Some precursor metabolites from intestinal bacteria may affect glutathione (GSH) levels.^33^ GSH administration attenuates CD95-mediated hepatic apoptosis and injury in mice.^34^ The metabolites, and the intestinal bacteria that produce them, play a vital role in the “liver-gut interaction” and need to be further explored.

The present study explored the association between liver damage and intestinal flora dysbiosis induced by vancomycin (Van) and gentamycin (Gen) in mice. We isolated and identified several differential strains from the gut bacterial populations of mice that received the two treatments. Further study showed that gavage with BA alleviated ConA-induced liver injury by downregulating hepatocyte CD95 expression in mice. This study demonstrated the protective effects of single microbial strains of BA on CD95-associated liver disease.

## Results

### Antibiotic-induced gut microbiota dysbiosis altered the susceptibility to ConA-induced liver inflammation

**Figure 1.**
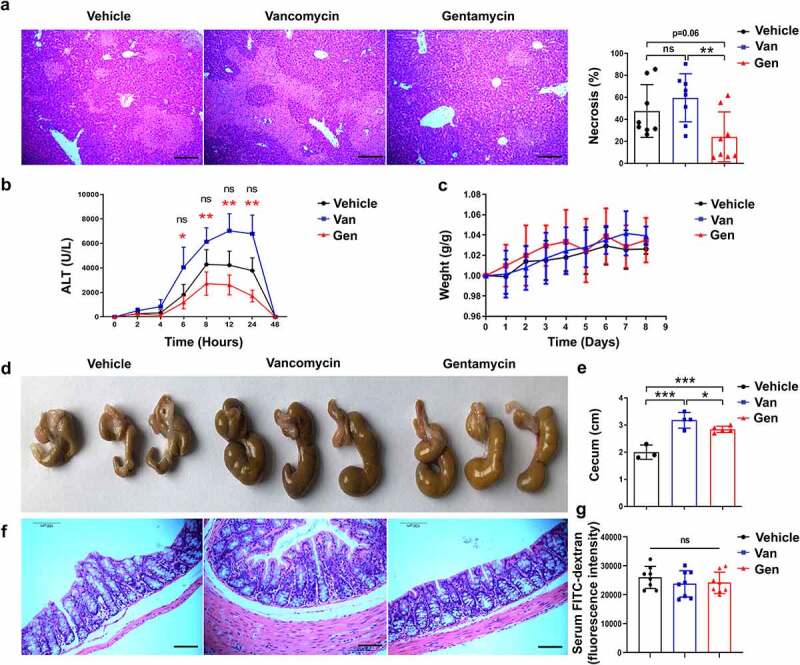
Male C57BL/6 mice treated with different antibiotics exhibited altered sensitivity to ConA-induced hepatitis. (a) H&E staining of liver sections and corresponding sizes of necrotic areas are shown from mice 12 h after ConA administration (n = 8 mice per group). (b) Serum ALT levels were measured at the indicated time points (n = 8 mice per group). (c) Body weight changes of mice after antibiotic treatment (n = 8 mice per group). (d, e) Representative image showing the mouse cecum (n = 3–5). (f) H&E-stained colonic tissue sections 8 h after ConA administration. (g) FITC-dextran fluorescence intensity in the serum of mice (n = 8 mice per group). Short horizontal lines indicate means. The data from two independent experiments were combined. *Black represents Vehicle vs. Gen. *Red represents Van vs. Gen. *P < .05, **P < .01, ***P < .001. ns, not significant (t test). Scale bars, 100 μm (a, f).

To directly assess the impact of the gut microbiota on the immune response in the liver, the effects of several antibiotics on liver homeostasis in mice were examined. We found that mice continuously pretreated with Van or Gen for 1 week had significantly altered susceptibility to ConA-induced liver injury. Twelve hours after intravenous ConA injection, the Van-treated mice exhibited significant liver damage, as evidenced by elevated serum alanine transaminase (ALT) levels and increased necrotic areas (Figure 1a,b). However, Gen treatment alleviated the elevation in serum ALT levels, and the livers of the Gen-treated mice exhibited much less necrosis than the Van-treated mice. Van and Gen treatments without ConA injection did not change the ALT levels or body weight compared with the vehicle group (Figure 1c, Supplementary Figure S1). The cecum of the mice treated with Van or Gen were larger and filled with contents of different colors (Figure 1d,e). The Gen-treated mice showed no significant difference in colonic mucosal integrity compared with the Van-treated or vehicle-treated mice (Figure 1f). The serum FITC-dextran assay also showed similar intestinal permeability in Gen-treated and Van-treated mice without ConA challenge (Figure 1g).

### Antibiotic treatment reshaped the murine microbial community

**Figure 2.**
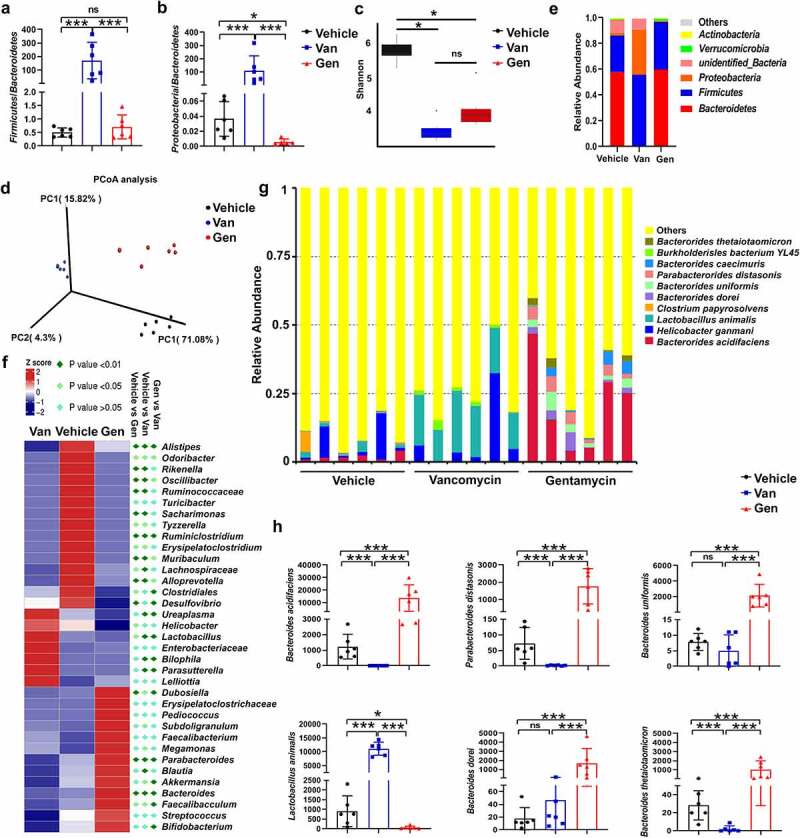
Antibiotic treatment altered the composition of the gut microbiota. 16S rRNA sequencing analysis of fecal DNA isolated from mice treated with antibiotics was performed (n = 6–8 mice per group). (a, b) *Firmicutes*/*Bacteroidetes* ratio and *Proteobacteria*/*Bacteroidetes* ratio. (c) Alpha diversity analysis. (d) Principal coordinates analysis. (e) Relative abundance of significantly altered taxa at the phylum level, including unspecified taxa. (f) Heatmap of the relative abundance of significantly altered taxa at the genus level. (g) Relative abundance of significantly altered taxa at the species level, including unspecified taxa. (h) Species with significantly increased abundance in Gen-treated mice (top 5) and Van-treated mice (top 1) are shown. The bars represent the means ± s.d. Statistical analyses: t test. *P < .05, ***P < .001. ns, not significant. See also Supplementary Figure S2.

To study the composition of the flora after antibiotic treatment, fecal DNA isolated from mice treated with antibiotics was analyzed using 16S rRNA sequencing. The analysis confirmed that Van and Gen treatment resulted in two distinct intestinal flora structures. The *Firmicutes/Bacteroidetes* and *Proteobacteria/Bacteroidetes* ratios were significantly increased in the Van-treated mice (Figure 2a,b). Alpha diversity analysis revealed that antibiotic treatment caused a significant decrease in species diversity (Figure 2c). Principal coordinates analysis also revealed the separation of three clusters (Figure 2d). At the phylum level, Van treatment increased the abundance of *Proteobacteria* and *Firmicutes* and decreased the abundance of *Bacteroidetes* in mice (Figure 2e), and the ratio of Gram-negative/positive bacteria did not significantly change (Supplementary Figure S2). At the genus level, the abundances of *Lactobacillus, Bilophila, Parasutterella* and *Ureaplasma* were enriched in the Van-treated mice, and *Bacteroides, Dubosiella* and *Parabacteroides* were enriched in the Gen-treated mice (Figure 2f). Among the 875 operational taxonomic units (OTUs) identified at the species level, the frequencies of the top 10 OTUs were significantly different between mice treated with Van and mice treated with Gen (Figure 2g). Six single species with relatively high proportions were counted (Figure 2h).

### The gut microbiota modulated the susceptibility to ConA-induced liver injury

**Figure 3.**
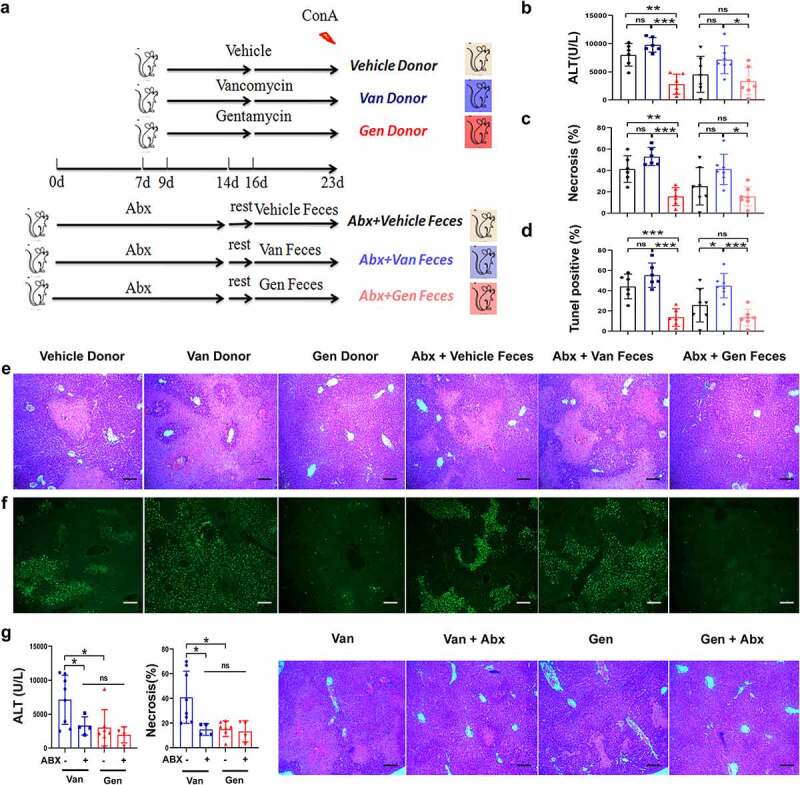
Susceptibility to ConA-induced liver injury was regulated by symbiotic bacteria. (a) Experimental strategy. (b) Microbiota-depleted recipient mice were transplanted with feces from donor mice. Serum ALT levels were measured 12 h after ConA administration (n = 6–8 mice per group). (c, d, e, f) Representative H&E and TUNEL staining of liver sections and corresponding sizes of necrotic areas from mice 12 h after ConA administration are shown (n = 6–8 mice per group). (g) Serum ALT levels and H&E staining necrosis of liver sections from Van- and Gen-treated mice treated with or without Abx 12 h after ConA administration (n = 5–8 mice per group). Statistical analyses: Student’s unpaired t test. *P < .05, ***P < .001. ns, not significant. Scale bars, 200 μm (e), 100 μm (f), 200 μm (g).

Wild-type mice were treated with a cocktail of antibiotics (Abx) to clear the intestinal flora. The mice rested in a squirrel cage for 2 days, and clean litter and sterile water were regularly provided. Sterile water was continuously provided. Feces were collected from vehicle mice and mice treated with individual antibiotics and transferred to Abx-pretreated mice (Figure 3a). Sensitivity to ConA-induced liver injury was assessed, and the sensitivity of the Abx-pretreated mice that received feces from the donor mice was consistent with the donor mice (Figure 3b,c,e). The Gen-treated and Gen fecal reconstituted mice exhibited only sporadic apoptotic cells, and more extensive apoptosis was observed in the Van-treated and Van fecal-reconstituted mice (Figure 3d,f). This result showed that susceptibility to hepatitis in mice with different gut microbiota was transferable via fecal transplantation. Abx treatment eliminated the difference in susceptibility to ConA-induced liver injury between Van- and Gen-treated mice (Figure 3g), which indicated an association between the gut microbiota and liver injury phenotype. Taken together, these data demonstrated that antibiotic-induced gut microbiota dysbiosis modified the susceptibility to ConA-induced liver injury in mice. Manipulation of the intestinal flora altered this susceptibility, as shown by the results of microbiota reconstitution.

### The difference in ConA-induced liver injury after Van and Gen treatment was independent of IFN-γ

**Figure 4.**
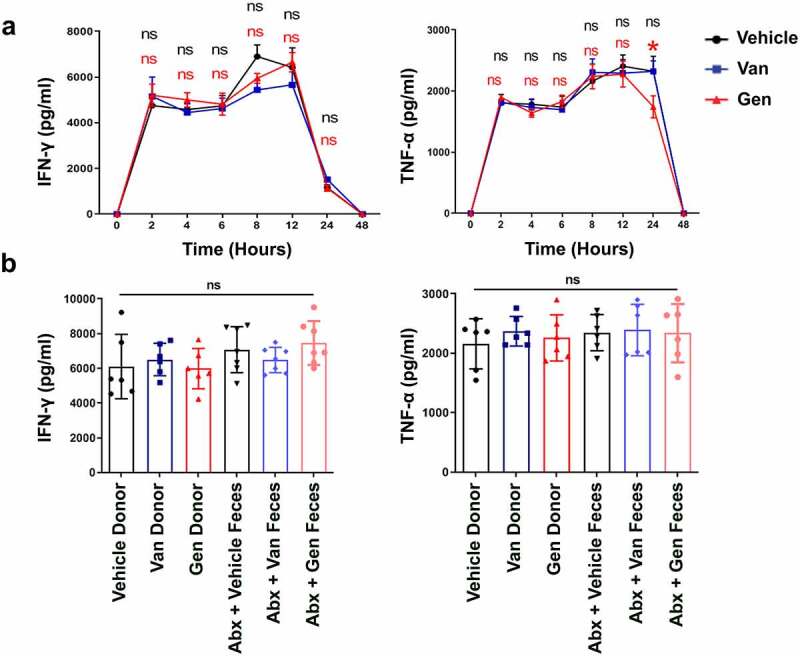
The difference in liver injury of gut microbiota-reconstituted mice was independent of IFN-γ and TNF-α. (a) IFN-γ and TNF-α cytokine profiles in the serum of Van- or Gen-treated mice 2 h, 4 h, 6 h, 8 h, 12 h, and 24 h after ConA injection (n = 8 mice per group). (b) Abx-treated recipient mice were transplanted with fecal bacteria from donor mice. IFN-γ and TNF-α levels were measured 12 h after ConA administration (n = 6–8 mice per group). ns, not significant (t test).

ConA-induced liver damage is predominantly mediated by natural killer T (NKT) cell-derived IFN-γ.^35^ The IFN-γ produced by CD4^+^ T cells also affects the severity of liver damage.^36^ Whether a significant difference in the number or composition of liver-resident immune cells occurred after antibiotic treatment was examined. There were similar frequencies of CD4^+^Foxp3^+^ Treg lymphocytes and CD11b^+^Gr1^+^ myeloid-derived suppressor cells (MDSCs) cells after antibiotic treatment (Supplementary Figure S4a,b). The results also showed no statistically significant difference in the frequency of NKT cells (Supplementary Figure S4c). Two and 12 h after ConA administration, there were no statistically significant difference in the levels of IFN-γ secreted by NKT cells after antibiotic treatment (Supplementary Figure S4d,e). The levels of IFN-γ produced by CD4^+^ T cells and total lymphocytes were also unchanged (Supplementary Figure S4f,g). The IFN-γ and tumor necrosis factor-α (TNF-α) production profiles were examined. Serum IFN-γ and TNF-α levels increased rapidly after ConA administration, but no difference was observed between the two antibiotic-treated and vehicle groups (Figure 4a). All groups of mice reconstituted with intestinal flora had similar serum IFN-γ and TNF-α levels 12 h after ConA administration (Figure 4b). Because of the overall similarity in cell composition after antibiotic treatment, it seems unlikely cell composition underlies that the difference in sensitivity to ConA in mice. These data suggest that differences in IFN-γ and TNF-α cytokine secretion do not explain the differential damage observed in the mice.

### The gut microbiota modulated hepatocyte apoptosis

**Figure 5.**
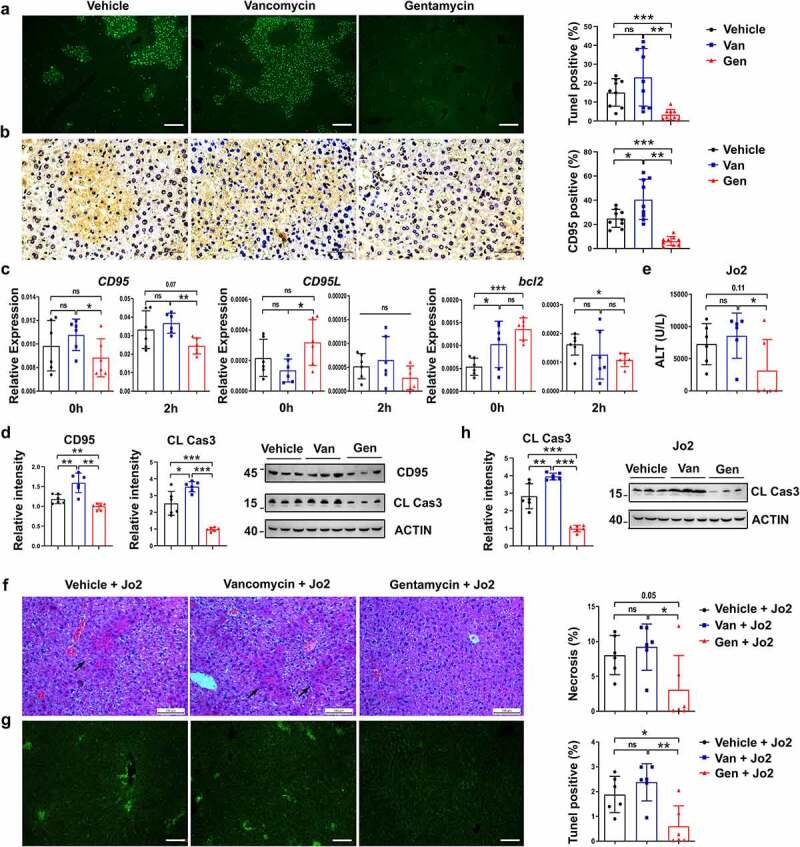
Van- and Gen-induced sensitivity alterations to liver injury in mice were CD95-mediated apoptosis dependent. TUNEL assay (a) and IHC staining of CD95 (b) in liver sections 12 h after ConA injection (n = 9 mice per group). (c) Relative expression of *CD95, CD95L* and *Bcl2* in livers 0 h (before ConA administration) and 2 h after ConA administration (n = 6–8 mice per group). (d) CD95 and cleaved caspase-3 levels in the liver 8 h after ConA administration (n = 6 mice per group). (e) Mice were injected with anti-CD95 (Jo2) at a dose of 0.2 µg g^−1^. Blood was harvested 12 h later for ALT level measurement (n = 7 mice per group). H&E staining (f) and TUNEL assay (g) of liver tissues, and cleaved caspase-3 expression (h) in liver sections 8 h after Jo2 administration are shown (n = 6 mice per group). Statistical analyses: Student’s unpaired t test. *P < .05, **P < .01. ns, not significant. Scale bars, 100 μm (a), 50 μm (b), 100 μm (f), 100 μm (g).

To explore the mechanisms underlying the effects of the different antibiotic treatments on hepatocytes, hepatocellular apoptosis was assessed after ConA administration. We found that the Gen-treated mice exhibited only sporadic apoptotic cells, and more extensive apoptosis was observed in the Van-treated mice (Figure 5a). Hepatocytes constitutively express the CD95 death receptor, and its cross-linking leads to hepatocyte apoptosis and acute liver injury.^37^ Therefore, whether the expression of the *CD95* gene was altered became a point of interest. The CD95 expression of hepatocytes in the Gen-treated mice was lower than the Van-treated mice (Figure 5b). CD95 transcript and protein levels in the liver tissues from the Gen-treated mice treated with or without ConA were significantly lower than the liver tissues from the Van-treated mice, and the expression of genes involved in apoptosis, including *Bcl2* and *CD95L*, was unchanged (Figure 5c,d). Gen treatment significantly reduced the level of ConA-induced liver apoptosis compared to Van treatment (Figure 5d). We hypothesized that the activation of CD95 signaling differed in the Van- and Gen-treated mice. Subsequent experiments induced liver damage using an agonistic monoclonal antibody (mAb) of CD95, Jo2, which directly activates the CD95 pathway and causes acute hepatocyte apoptosis.^38^ After Jo2 treatment, differences in the susceptibility to liver injury and apoptosis were observed in the Van- and Gen-treated mice (Figure 5e,f,g). The level of cleaved caspase-3 was lower in Gen-treated mice than Van-treated mice with Jo2-induced liver injury (Figure 5h). We confirmed that antibiotic treatment altered hepatocyte CD95 expression and affected ConA-induced liver damage.

### Pretreatment with BA regulated the sensitivity to ConA-induced liver injury by inhibiting CD95-dependent hepatocyte apoptosis

**Figure 6.**
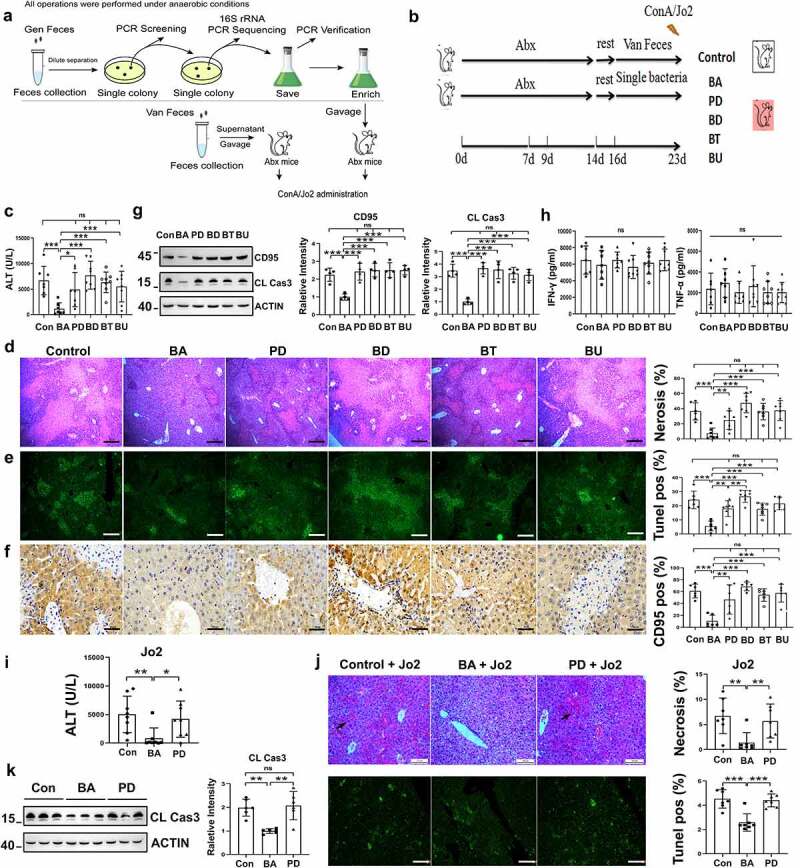
*B. acidifaciens* reconstitution resulted in lower sensitivity to ConA- and Jo2- induced liver injury in Abx-pretreated mice. (a, b) Experimental strategy. (c) Serum ALT levels were measured 12 h after ConA injection. H&E (d), TUNEL (e) and CD95 IHC staining (f) of liver sections of Abx-pretreated mice reconstituted with a single bacterial strain and treated with ConA (12 h) (n = 6–8 mice per group). (g) CD95 and cleaved caspase-3 expression levels of Abx-pretreated mice reconstituted with a single bacterial strain (isolated from mice) 8 h after ConA injection (n = 6–8 mice per group). (h) Serum IFN-γ, and TNF-α levels were measured 12 h after ConA injection. Serum ALT levels (i) H&E staining and TUNEL assay (j) of BA- and PD-transferred Abx-pretreated mice 12 h after the administration of 0.2 µg g^−1^ anti-CD95 (Jo2) (n = 8 mice per group). (k) Cleaved caspase-3 expression levels in the liver measured 8 h after Jo2 administration are shown (n = 6 mice per group). The data from three independent experiments were combined. Statistical analyses: Student’s unpaired t test. *P < .05, ***P < .001. ns, not significant. Scale bars, 50 μm (d), 200 μm (e), 50 μm (f), 100 μm (j).

We explored the potential mechanism of gut microbiota regulation of ConA-induced liver injury. BA, *Parabacteroides distasonis* (PD), *Bacteroides thetaiotaomicron* (BT), *Bacteroides dorei* (BD) and *Bacteroides uniformis* (BU) were the dominant bacteria in the Gen-treated mice, and their enrichment was negatively associated with serum ALT levels (Supplementary Figure S3). These five single bacterial strains were isolated from the feces of mice that consumed Gen (Figure 6a). Abx-pretreated mice were administered BA, PD, BT, BD or BU (Figure 6b). BA-reconstituted mice pretreated with Abx were more resistant to ConA-induced liver injury than mice reconstituted with the other four bacterial strains (Figure 6c). Treatment with BA alleviated liver necrosis (Figure 6d) and hepatocyte apoptosis (Figure 6e) compared to the other four strains. CD95 and cleaved caspase-3 levels in the liver were also reduced in BA-reconstituted mice (Figure 6f,g). However, no considerable difference in serum IFN-γ or TNF-α levels was observed between mice treated with each bacterial strain (Figure 6h). Liver injury and hepatocyte apoptosis were alleviated in the BA-reconstituted mice after Jo2 injection (Figure 6i,j), which indicated that the amelioration of the inflammatory state depended on CD95 (Figure 6k). Collectively, these results indicated that BA modulated liver injury by suppressing CD95-dependent hepatocyte apoptosis.

### Pretreatment with BA reduced sensitivity to alcohol-induced liver injury by suppressing hepatocyte apoptosis

**Figure 7.**
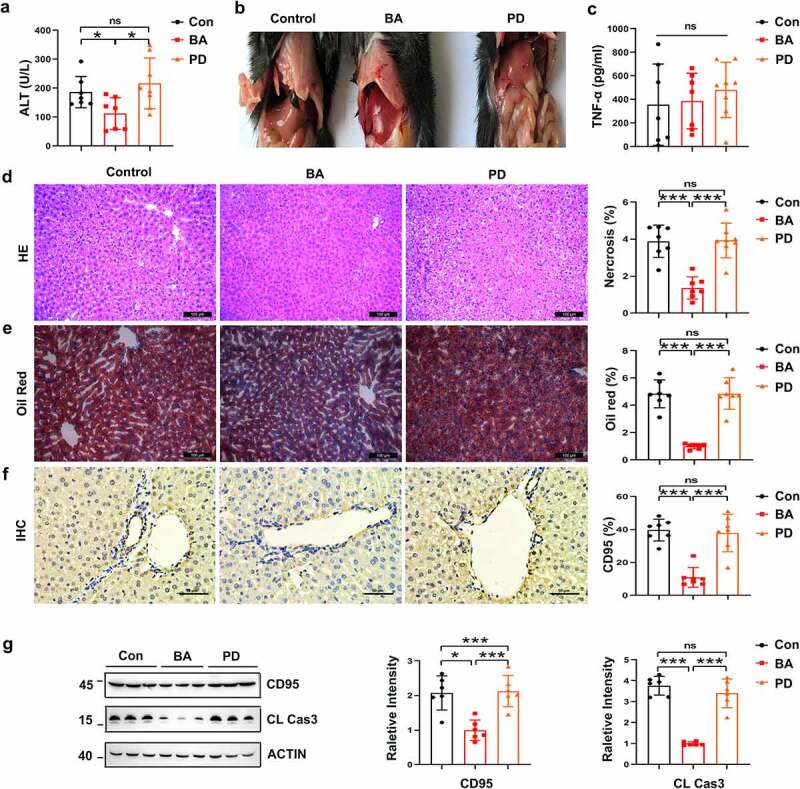
*B. acidifaciens* reconstitution resulted in lower sensitivity to alcohol-induced liver injury. Serum ALT (a) and TNF-α levels (c) were measured 12 h after alcohol treatment (6 g kg^−1^) (n = 8 mice per group). (b) Gross liver appearance at 12 h. Representative H&E (d), Oil red O (e) and CD95 IHC staining (f) of liver sections from mice 12 h after alcohol treatment (n = 6–8 mice per group). (g) CD95 and cleaved caspase-3 expression in the liver 8 h after alcohol administration (n = 6 mice per group). Scale bars, 100 μm (d, e). 50 μm (f). *P < .05, ***P < .001. ns, not significant (t test).

CD95-mediated hepatocyte apoptosis is also involved in alcohol-induced liver injury. BA-reconstituted mice exhibited lower serum ALT levels (Figure 7a) than the control or PD-reconstituted mice, and serum TNF-α levels were consistent in the three groups after alcohol treatment (Figure 7c). Liver tissues from the BA-reconstituted mice showed slight necrosis and vacuolar degeneration compared with the control mice (Figure 7b,d). Alcohol-induced lipid droplet accumulation was also significantly decreased (Figure 7e). CD95 expression in the liver tissue was reduced after alcohol administration, as shown by immunohistochemical staining and immunoblotting (Figure 7f,g). Hepatic cleaved caspase-3 levels were also reduced (Figure 7g). Therefore, BA pretreatment ameliorated alcoholic liver injury in mice in a CD95-dependent manner.

### Pretreatment with BA decreased the liver L-glutathione/glutathione (GSSG/GSH) ratio, which played a critical role in reducing CD95 expression

**Figure 8.**
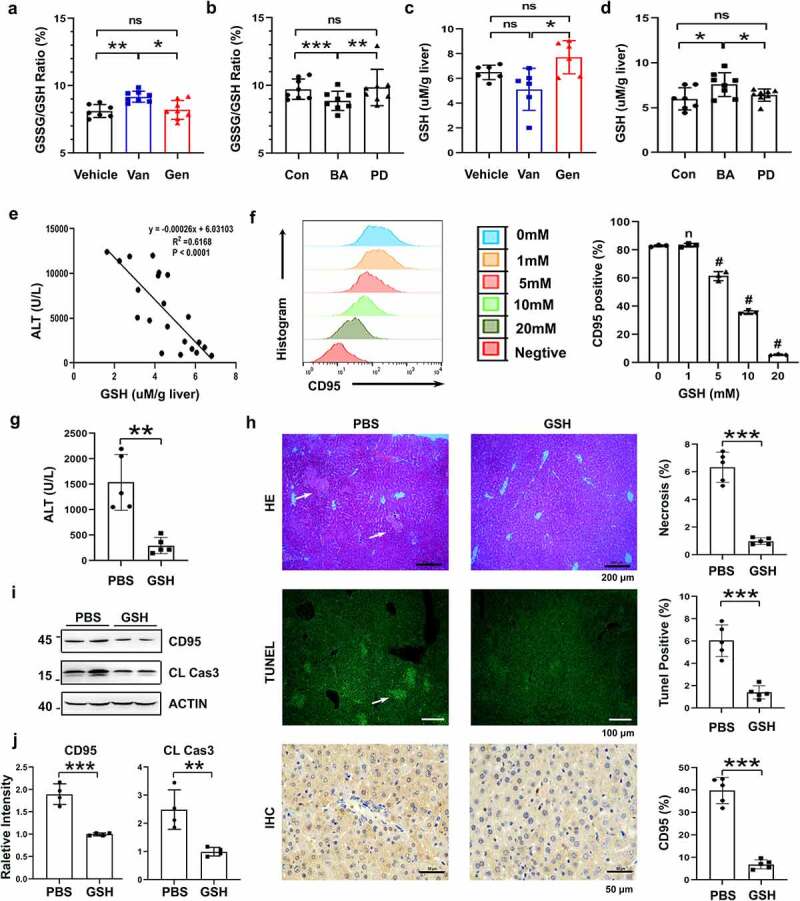
GSH suppressed CD95 expression and ConA-induced liver damage. Liver GSSG/GSH ratio of Van- and Gen-treated mice (a) and BA- and PD-transferred Abx-pretreated mice (b) (n = 7–8 mice per group). Liver GSH levels of Van- or Gen-treated mice (c) and BA- and PD-transferred Abx-pretreated mice (d) 12 h after ConA-injection (n = 6–8 mice per group). (e) The relationship between serum ALT levels and liver GSH levels after ConA administration was analyzed (n = 21 mice). (f) Hepa1–6 cells were cocultured with GSH for 24 h, and CD95-positive cells were quantified using FACS (n = 3 independent biological replicates/group). (g) Serum ALT levels assay of GSH-treated mice (40 mg mice^−1^) 12 h after ConA injection (n = 5 mice per group). (h) H&E, TUNEL assay and IHC staining of CD95 in liver sections are shown. (i. j) CD95 and cleaved caspase-3 expression in the liver 8 h after GSH administration (n = 4 mice per group). Statistical analyses: Student’s unpaired t test. n, Not different from the Control (0 mM), #, *P* < .001. ***P < .001, *P < .05, **P < .01, ns, not significant.

No significant difference in colonic mucosal integrity or intestinal permeability was detected among the BA-treated, PD-treated and control mice in histopathological staining and serum FITC-dextran assay of colon tissue (Supplementary Figure S6a,b). No bacteria were detected in the 16S rRNA analysis of liver tissue and culture of the liver homogenate, which indicates that no intestinal bacterial translocation occurred after antibiotic treatment in this study. Therefore, neither antibiotics nor BA treatment affected the intestinal barrier function of mice. We hypothesized that intestinal BA affected CD95 expression by producing metabolites. Previous studies showed that bile acids, SCFAs, and glutathione were involved in hepatocyte apoptosis.^34,39,40^ The present study performed a metabonomic analysis of SCFAs and bile acids in the livers of Van- and Gen-treated mice (Supplementary Figures S7, S8). The levels of some SCFAs and bile acids in the livers of the Gen-treated mice were significantly higher than the Van-treated mice (Supplementary Figures S7c and S8). However, subsequent studies showed that these metabolites did not significantly inhibit CD95 expression and liver injury in vitro and in vivo (Supplementary Figure S9a-f). Notably, the liver GSSG/GSH ratio was significantly decreased in the mice undergoing Gen or BA treatment (Figure 8a,b). The GSH levels in the liver were also increased in the Gen-treated or BA-reconstituted mice after ConA injection (Figure 8c,d). Serum ALT levels negatively correlated with hepatic GSH levels in the experimental mice (Figure 8e). Therefore, the GSH levels and GSSG/GSH ratio in the liver were likely involved in suppressing CD95 expression and the protective effect of BA on liver injury. We observed reduced CD95 expression in Hepa1–6 cells stimulated with GSH (Figure 8f). Treatment with GSH eliminated the increase in serum ALT levels after ConA injection (Figure 8g). Livers of the GSH-treated mice exhibited substantially reduced necrosis, apoptosis and CD95 expression compared to the control mice (Figure 8h,i,j). Overall, these results suggest that BA exerts a protective role in CD95-mediated mouse liver injury by decreasing the GSSG/GSH ratio in the liver.

**Figure 9.**
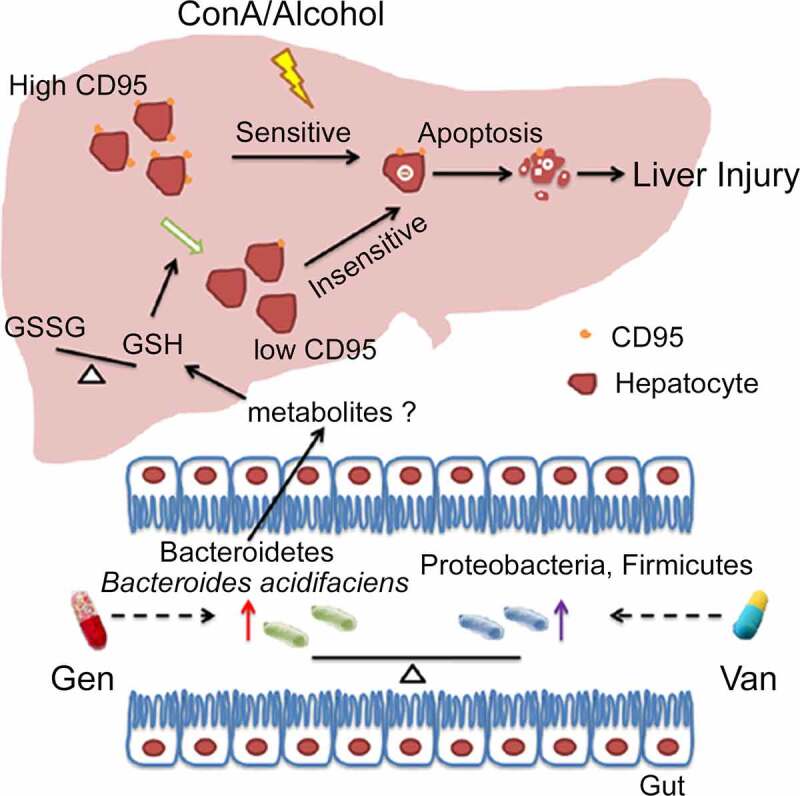
Working model of the mechanism of BA protection against CD95-dependent autoimmune/alcohol-induced liver injury. Antibiotics induce polarization of the gut microbiota. Gen treatment induces the enrichment of BA in the intestine. BA decreases the GSSG/GSH ratio in the liver, likely by secreting some metabolites that inhibit hepatocyte apoptosis and ConA- or alcohol-induced liver injury by suppressing CD95 expression in hepatocytes.

## Discussion

The gut microbiota plays an important regulatory role in many human and mouse liver diseases.^41,42^ Differences in intestinal flora composition may lead to diversities in the severity of liver diseases in the host.^14^ Previous studies identified some bacterial genera that were relevant to autoimmune or alcoholic hepatitis in humans and animals using correlation analysis, such as *Faecalibacterium, Veillonella, Akkmensia, Blautia, Parabacteroides* and *Bacteroides*.^21,32,43–45^ The regulatory functions of several intestinal bacterial species, such as *Akkmensia muciniphila* and *Lactobacillus rhamnosus*, in autoimmune and alcoholic liver injury were identified.^46,47^ However, only a few species have explicit regulatory effects on autoimmune or alcoholic liver injury among the thousands of species of intestinal bacteria. Therefore, we used antibiotic-induced intestinal dysbiosis in mice combined with a ConA-induced autoimmune hepatitis mouse model to discover an intestinal *Bacteroides* species that plays a protective role in the liver. *Bacteroides* have gained increasing attention because of their potential regulatory roles in liver diseases, such as NASH and alcoholic hepatitis.^48,49^ Mice from the Jackson Laboratory (JAX) are more sensitive to ConA-induced liver injury than mice from Taconic Farms (TAC). The abundance of intestinal *Bacteroides* in JAX mice is significantly lower than TAC mice.^14^ Gut *Bacteroides* is also more abundant in alcohol-resistant mice than alcohol-sensitive mice. Pectin administration significantly alleviated alcohol-induced liver damage and improved the abundance of *Bacteroides*.^22,50^ However, there is little knowledge of the specific *Bacteroides* species that play regulatory roles in autoimmune and alcoholic hepatitis and their functional mechanism. We demonstrated the protective mechanism against autoimmune and alcoholic liver injury of the *Bacteroides* species.

The present study used Gen and Van to induce intestinal flora dysbiosis in mice followed by a ConA-induced liver injury mouse model. We found that the abundance of four *Bacteroides* species, BA, BU, BT, BD, and 1 *Parabacteroides* species, PD, negatively correlated with the damage level of the liver by comparing the intestinal flora composition formed after the two antibiotic treatments. We isolated and focused on the potential protective effects and mechanisms of the five species on liver injury. We first report *Bacteroides acidifaciens* as a critical intestinal bacterial species that plays a protective role against ConA- and alcohol-induced liver injury via functional exploration in vivo. We demonstrated that BA administration decreased the GSSG/GSH ratio, downregulated CD95 expression in hepatocytes in the liver and inhibited hepatocyte apoptosis, which protected the liver from severe damage. Our results provide evidence of BA regulatory function in the “gut-liver axis”.

Differentiated gut microbiota compositions are often used to study the regulatory function of intestinal flora. Previous studies showed that a variety of food or drug components, such as pectin,^22,50^ a high-fiber diet,^51^ a high-fat diet,^52^ emodin,^53^ quercetin^54^ and various antibiotics,^55^ changed the composition of the intestinal flora and affected its function. Artificially induced intestinal flora dysbiosis using antibiotics in healthy mice raised in the same living environment generated large differences in gut microbiota due to the distinct antimicrobial spectrum, which likely produces considerable differences in pathological responses. Antibiotic-induced gut microbiota dysbiosis is also conducive to the subsequent bacterial reconstitution, enrichment, isolation and functional identification of certain target bacterial species. The present study used two nonabsorbable antibiotics with distinct antimicrobial spectra, Van and Gen, to induce intestinal flora dysbiosis in mice. Intragastric administration of Van and Gen in mice generated two distinct compositions of intestinal flora and significantly different sensitivities to ConA-induced liver injury. Fecal microbiota transplantation (FMT) indicated that the diverse composition of the intestinal microbiota caused differences in the degree of liver injury. *Firmicutes* and *Proteobacteria* were the dominant microbes at the phylum level in the intestinal flora of mice after Van treatment in the present study, and *Verrucomicrobia* was rare. However, previous studies showed that *Verrucomicrobia, Firmicutes* and *Proteobacteria* were the predominant bacteria in Van-treated mice.^56,57^ At the genus level, *Lactobacillus* was the dominant genus of the intestinal flora, and *Akkermansia* was present in a small proportion after seven days of Van treatment in the present study, which was the opposite of previous studies.^57^ This result may be due to the diverse composition of the initial gut microbiota in mice, which results in partially different consequences on intestinal flora structure after the same antibiotic treatment. A “gut microbiota-disease correlation analysis” was performed to identify many intestinal bacteria that were strongly associated with common liver diseases, such as autoimmune liver injury, viral hepatitis, and alcohol-induced liver injury.^14,58^ However, this “gut microbiota-disease correlation analysis” could not precisely confirm the causality between the diseases and the intestinal bacteria. Further functional studies discovering and elaborating the mechanism of the intestinal flora are indispensable. Our studies identified that the abundance of five bacterial species, BA, BU, PD, BT and BD, significantly negatively correlated with the level of hepatic damage using gut microbiota-liver injury level correlation analysis. None of these five species were previously associated with autoimmune hepatitis in humans or mice. However, subsequent functional verification of single bacterium reconstitutions in vivo showed that only the relevance between BA, but not BU, PD, BT or BD, and liver injury level was consistent with its protective function in vivo. Therefore, a large gap may exist between the predicted function based on a correlation analysis of the intestinal flora and their real biological function in vivo. However, these bacterial species may function synergistically with other intestinal bacterial species to ameliorate liver damage. This hypothesis requires further analysis and experimental verification.

ConA-induced liver injury is a T cell-mediated and IFN-γ-dependent autoimmune liver disease in mice.^35^ Previous studies showed that NKT and CD4 + T cells aggravated ConA-induced liver injury by producing IFN-γ.^36,59^ However, our data showed that the difference in ConA-induced liver injury caused by Gen and Van treatment was independent of IFN-γ, NKT and CD4 + T cells. The liver is a “CD95-sensitive” organ in which various cells express CD95 on their surfaces.^60^ CD95-mediated apoptosis is crucially involved in autoimmune liver, alcoholic hepatitis, viral hepatitis and other liver diseases.^61^ The CD95 agonistic antibody Jo2 activates CD95-dependent apoptosis signals and directly induces liver injury. Previous studies showed that the gut microbiota of JAX mice aggravated Jo2-induced acute liver injury compared to TAC mice.^14^ This result indicated that intestinal flora regulated liver injury by modulating CD95 expression. The present study confirmed the inhibitory effect of Gen administration and BA reconstitution on CD95 expression in the mouse liver by detecting CD95 expression (quantitative PCR, Western blot (WB) and immunohistochemistry (IHC)) in vitro and Jo2-induced liver injury in vivo. These results suggested that intestinal BA was a key species in the regulation of CD95 expression in the liver. This result is consistent with previous studies that showed a negative correlation between the abundance of intestinal *Bacteroides* and CD95 expression in the liver.^14^ Considering the function of CD95 in aggravating alcoholic liver injury and the universality of alcoholic hepatitis,^9^ we demonstrated that BA also protected the mouse liver from an alcohol-induced liver injury model by inhibiting CD95 expression. More exploration is needed to confirm whether BA regulated other liver diseases by inhibiting hepatic CD95 expression.

Previous studies showed that *Bacteroides* protected intestinal barrier function, which had an important influence on liver diseases.^42^ HE staining of colonic tissue and FITC-dextran fluorescence detection in the serum indicated that antibiotic treatment or BA and PD intragastric administration did not change intestinal barrier integrity or permeability in the present study. No bacteria were detected in the 16S rRNA analysis of the mouse liver tissue and liver homogenate culture after antibiotic treatment or BA and PD gavage. These results suggested that some metabolites secreted by BA, instead of bacterial cells, may enter the liver through the portal vein to play a protective function. Compelling evidence revealed that a variety of bioactive metabolites generated by the intestinal flora exerted protective functions in the liver, such as SCFAs and bile acids.^39,62–66^ Previous studies reported that chenodeoxycholic acid (CDCA) improved ConA-induced liver injury and downregulated CD95 expression.^39^ We performed metabolome analysis of bile acids and SCFAs in the livers of antibiotic-treated mice. There was no correlation between the relative content of CDCA and liver injury level, which is inconsistent with a previous report.^39^ Some bile acids and SCFAs in Gen-treated mice were significantly higher than Van-treated mice, such as glycocholic acid (GCA), propionic acid and butyric acid. However, these metabolites did not show CD95 inhibition or protection against ConA-induced liver injury in vitro or in vivo. GSH content in the liver negatively correlates with ConA-induced liver injury.^67^ A previous study confirmed that GSH effectively suppressed CD95-mediated cell apoptosis.^34^ GSH is directly or indirectly synthesized by intestinal bacteria or the liver itself.^33,68–70^ The present study found that BA administration in mice decreased the hepatic GSSG/GSH ratio. GSH also significantly inhibited CD95 expression and alleviated ConA-induced liver injury in vitro and in vivo. We hypothesized that some metabolites secreted by BA entered the liver through the portal vein and decreased the GSSG/GSH ratio, which suppressed CD95 expression. Unfortunately, we could not identify the key metabolite from BA that regulated the GSSG/GSH ratio and CD95 expression. Intestinal bacteria may produce diverse types and levels of metabolites under different living environments and nutritional conditions. Therefore, it is challenging to establish suitable culture conditions in vitro that reproduce intestinal bacteria functions in vivo. Further studies of the regulatory effects of these intestinal flora-derived metabolites in the liver will help improve our understanding of the regulatory mechanisms of the “liver-gut axis”.

In conclusion, our findings help elucidate the functional mechanisms and consequences of gut-liver interactions. We established *Bacteroides acidifaciens* as a critical intestinal bacterial species for liver homeostasis (Figure 9). Because CD95-mediated hepatocyte apoptosis is widely involved in various types of acute and chronic hepatic injuries, BA may hold promise as a potent hepatoprotective agent in CD95-associated liver diseases.

## Materials and methods

### Mice

Male C57BL/6 mice for breeding were purchased from Vital River Laboratory Animal Technology Co., Ltd. All mice were fed under specific pathogen-free conditions for three generations in the Experimental Animal Center at Nankai University. The gut microbiota of 7-week-old male C57BL/6 mice was depleted (Abx group) using a cocktail of antibiotics. Neomycin sulfate (10 mg ml^−1^; Solarbio, China), ampicillin (10 mg ml^−1^; Roche), metronidazole (10 mg ml^−1^; Sigma-Aldrich), Van (5 mg ml^−1^; Sangon, China), and amphotericin B (0.1 mg ml^−1^; Sigma-Aldrich) were administered via gavage in a volume of 200 µl per mouse twice daily for two weeks. The clearance of the intestinal flora was verified using 16S rRNA sequencing analysis. Van (5 mg ml^−1^; Van group), Gen (5 mg ml^−1^; Gen group, Sangon, China), or an equal volume of sterile water (Vehicle group) was administered via gavage in a volume of 200 µl per mouse twice per daily for one week. All mice (four mice per cage) were given free access to water and food and maintained in a temperature-controlled (22°C) room on a 12:12-h light/dark cycle (8:00 AM, lights on; 8:00 PM, lights off). The Nankai University Experimental Animal Ethics Committee approved all animal procedures.

### Induction and assessment of hepatitis

ConA (10 mg kg^−1,^ Sigma-Aldrich) diluted in phosphate-buffered saline was injected into C57BL/6 mice via the tail vein to induce hepatitis. CD95-mediated liver injury was induced via intraperitoneal injection of 0.2 μg g^−1^ of the anti-CD95 mAb Jo2 (BD Biosciences) into mice. Ethanol-induced liver injury was generated by administering 50% ethanol (6 g kg^−1^) via gavage. Liver injury was quantified by measuring serum alanine aminotransferase (ALT) levels (12 h after ConA/Jo2/ethanol treatments) and performing statistical analyses of histological staining (8 h or 12 h after ConA/Jo2/ethanol treatments). Liver tissues (100 mg) were harvested from mice for immunoblotting 8 h after ConA/Jo2/ethanol treatment. Liver tissues were harvested from mice and fixed in 4% paraformaldehyde (the same portion of liver tissue was harvested from each mouse), and 4-mm-thick sections of paraffin-embedded tissues were prepared. The samples were subjected to hematoxylin and eosin (H&E) and IHC staining, and the area of liver necrosis was quantified. Hepatocyte apoptosis was detected using TUNEL assays with a One Step TUNEL Apoptosis Assay Kit (Beyotime, C1088, China) according to the manufacturer’s instructions, and immunoblotting. Serum cytokine levels (2 h, 4 h, 6 h, 8 h, 12 h, and 24 h) were detected using ELISAs according to the manufacturer’s instructions. The GSH and GSSG levels in the liver homogenates were measured 0 and 12 h after ConA administration using a GSH and GSSG Assay Kit (Beyotime, S0053, China) according to the manufacturer’s instructions.

### Reagents

ConA (Sigma Aldrich), Jo2 (BD Biosciences), PE-conjugated CD11b antibody (clone M1/70), APC-conjugated Gr-1 antibody (clone RB68C5), PE-conjugated CD95 antibody (clone SA367H8), PE-conjugated NK1.1 antibody (clone PK136), FITC-conjugated TCR-β antibody (clone 1D11), APC-conjugated IFN-γ antibody (clone XMG1.2), and PE-conjugated CD4 antibody (clone 145–2C11) (BioLegend) were used. For IHC, tissue sections were stained with a CD95 antibody (Servicebio, China). PMA, ionomycin, and GolgiStop were purchased from BD Biosciences. Alanine aminotransferase kits were purchased from Shanghai Rongsheng Company. Mouse TNF-α (cat. 430905) and IFN-γ (cat. 430805) ELISA kits were purchased from BioLegend.

### Isolation of liver mononuclear cells (MNCs) and cell staining

Liver MNCs were isolated and purified using a previously described method, with some modifications.^59^ Briefly, homogenized liver cells were resuspended in 40% Percoll (GE Healthcare), gently overlaid onto 70% Percoll, and centrifuged for 30 min at 1,260 g. The purified MNCs were collected from the interface for further study. Surface staining was performed using the indicated fluorescently labeled Abs against surface proteins. For intracellular staining, liver MNCs were stimulated with PMA (50 ng ml^−1^) and ionomycin (1 µg ml^−1^) in the presence of GolgiStop for 5 h. The cells were fixed in 4% paraformaldehyde, permeabilized with 0.5% saponin (Sigma-Aldrich), and stained as previously described.

### RNA extraction and quantitative PCR analysis (qRT-PCR)

Total RNA was extracted from tissues using TRIzol Reagent from Life Technologies. qRT-PCR analyses of mRNA expression were performed using PrimeScript RT-PCR kits (Takara, Japan). The mRNA levels of β-actin were used as the internal control. Gene expression values were normalized to β-actin expression values. For the primer sequences, please refer to the Supplementary Information (Supplementary Table 5).

### Intestinal flora reorganization

To transplant intestinal microbiota, 150 mg fresh feces pellets were collected from donor mice after treatment with individual antibiotics for one week, and the pellets were homogenized in 3 ml sterile PBS under anaerobic conditions (on ice). The fecal pellets were homogenized and incubated at room temperature for 5 min, and 2 ml fecal supernatant was gavaged (200 μl per mouse) into microbiota-depleted mice twice daily for one week. For further details on the materials used, please refer to Figure 3a.

### Fecal DNA extraction and 16S quantification

Fecal samples (20 mg) were freshly collected from individual mice and frozen at −198°C until processing. DNA extraction was performed using the QIAamp DNA Stool Mini Kit (Qiagen, Germany). 16S gene quantification was performed with 10 ng fecal DNA and V3-V4 universal bacteria primers (338 F and 806 R). 16S rRNA deep sequencing, bioinformatics quality filtering, and OTU assignments were performed by Novegene Company. Statistical analyses were performed, and bacterial abundance and diversity were calculated.

### Bacterial strains, isolation, culture, preparation and transfer

Briefly, fecal contents were diluted in sterile PBS and inoculated on BHI agar plates (Qingdao Hope Biotechnology). The plates were anaerobically incubated (10% H_2_, 10% CO_2_, and 80% N_2_) at 37°C for 48 h (AW300SG anaerobic workstations; Electrotek, England). Colonies were selected and cultured under anaerobic conditions for separation (Figure 6a, Supplementary Figure S5). BA, BT, BU and BD were isolated. PD was isolated from the feces of mice as previously described.^71^ DNA was extracted and analyzed. For further details, please see Supplementary Tables 1–4, 6. The bacterial strains were cultured in anaerobically sterilized BLHK (BHI + (0.25 g/L) L-cysteine + (0.25 g/L) Na_2_S 9H_2_O + (5 mg/ml) hemin + (10 ug/ml) vitamin K1) medium under strict anaerobic conditions for 36 h for enrichment. The cultures were centrifuged at 9200 rpm for 5 min, washed with sterile PBS twice and resuspended in 2 ml sterile PBS containing 3 * 10^9 colony-forming units (CFUs). Live bacterial strains were gavaged into microbiota-depleted mice (200 μl per mouse) twice daily for one week. For further details on the materials used, please refer to Figure 6a,b and Supplementary Figure S5.

### Cell culture

Hepa1–6 cells were cultured in RPMI 1640 medium (10% FBS, 1% penicillin-streptomycin). 5 * 10^5 Hepa1–6 cells were cultured for 12 h before GSH/ metabolite treatment. Different concentrations of GSH/metabolite (dissolved in RPMI 1640) were added to the medium. After 24 h, CD95-positive cells were quantified using FACS.

### Intestinal permeability

FITC-dextran 3000–5000 Da (FD4, Sigma) was used to assess intestinal permeability in vivo. Mice were fasted for 5 h and orally gavaged with 15 mg of FITC-dextran (150 mg/ml). Five hours later, blood samples were obtained from mice. After two centrifugation steps at 12000 rpm for 3 min, serum FITC levels were determined using fluorometry at 490 nm with a microplate reader (Cytation5, Biotek, US).

### Immunoblotting

Liver tissue was harvested and lysed in RIPA lysis buffer (R0010, Solarbio) supplemented with 10 μM PMSF. Equivalent amounts of 100 µg of protein were separated on SDS-PAGE gels. The proteins were transferred to nitrocellulose membranes blocked with 5% skim milk and probed with the indicated primary antibodies, followed by the appropriate HRP-conjugated secondary antibodies. The following antibodies were used: anti-actin (1:3000, Sigma, A5441), anti-cleaved caspase-3 (1:1000, Cell Signaling Technology, 9664S), and anti-CD95 (1:1000, Abclonal, A0233).

### Bile acid analysis

Liver samples (100 mg) were resuspended in liquid nitrogen, homogenized with 900 μl water and centrifuged at 4,000 rpm for 10 min at 4°C. The supernatant was harvested and diluted it to five times the volume of acetonitrile/methanol (8:2) and centrifuged at 12000 rpm for 20 min to remove the protein. The supernatant was dried using a nitrogen blower, resuspended and centrifuged with 100 μl water/acetonitrile (8:2) and formic acid (0.1%). 2 μl supernatant was injected into an ultrahigh-performance liquid chromatography coupled to tandem mass spectrometry (UHPLC-MS/MS) system (ExionLC™ AD UHPLC-QTRAP® 6500+, AB SCIEX Corp, USA) for detection. Chromatographic separation was performed on an Acquity BEH C18 column (100 mm × 2.1 mm i.d., 1.7 μm), which was maintained at 50°C. The mobile phase consisted of 0.1% formic acid in water and acetonitrile. The flow rate was 0.30 ml/min. The solvent gradient was composed of the following components: initial 20% B, 0.5 min; 20–35% B, 1 min; 35–37% B, 2.5 min; 37–38% B, 4.1 min; 38–39% B, 6 min; 39–40% B, 6.5 min; 40–44% B, 8.5 min; 44–45% B, 9 min; 45–52% B, 9.5 min; 52–65% B, 11.5 min; 55–100% B, 12.5 min; 100–20% B, 15.1 min; and 20% B, 17 min. Gradient elution was applied, and MS detection proceeded in negative mode. A mass range of m/z 50 to 850 was acquired. Bile acid standards were used to identify the different bile acids detected by the UHPLC-MS/MS system.

### SCFAs analysis

Liver samples (100 mg) were homogenized with 100 μl 15% phosphoric acid, 100 μl isohexanoic acid solution (50 μg/ml) and 400 μl ether for 1 min, centrifuged at 12000 rpm at 4°C for 10 min, and the supernatant was obtained. Supernatant (1 μl) was injected into GC-MS (30 m × 0.25 mm i.d., 0.25 μm, Agilent) for testing. The split flow ratio was 10:1. The injector temperature was 250°C, the ion source temperature 230°C, the transmission line temperature 250°C, and quadrupole rod temperature 150°C. The programmed initial temperature was 90°C, which then increased 10°C/min temperature to 120°C. The temperature rose to 150°C at 5°C/min. The temperature was heated to 250°C at 25°C/min 2 min. The carrier gas was helium, and the carrier gas flow rate was 1.0 ml/min. The following MS conditions were used: electron bombardment ionization (EI) source, full scan and SIM scan mode, and electron energy 70 eV. SCFA standards were used to identify the different SCFAs detected by the GC-MS system.

### Statistics

Statistical analyses were performed using ImageJ, GraphPad Prism v8.3.1 (GraphPad Software) and R. The data are presented as the means ± SEM. An unpaired t test or Wilcoxon rank-sum test was used to analyze differences between two groups. (*Ρ < 0.05, **Ρ < 0.01, and ***Ρ < 0.001). All experiments were performed in at least two independent replicates. Spearman’s correlation tests were performed using the psych R package^72^ for multiple comparisons.

## Supplementary Material

Supplemental Material

## Data Availability

The 16S rRNA gene sequencing data were deposited in the NCBI database under accession code PRJNA752279. All data are available from the corresponding author upon reasonable request.
