# Supplementary material for: Bacteroides acidifaciens in the gut plays a protective role against CD95-mediated liver injury
Source: Gut Microbes. 2022 Feb 6;14(1):2027853. doi: 10.1080/19490976.2022.2027853 (PMC8820816; doi:10.1080/19490976.2022.2027853)
Supplement: Supplemental Material [file KGMI_A_2027853_SM7429.zip › supplementary/Supplementary Information.zip!/Supplementary Information/Supplementary figure legends.doc]

Figure S1: Van or Gen did not directly cause liver damage without ConA treatment. Male C57BL/6 mice were treated with Van or Gen for 1 week, and the serum ALT levels were measured before ConA administration (n = 8 mice per group). The data from three independent experiments were combined. **P < 0.01, ns: not significant (t test).

Figure S2: (a) The relative abundance of gram-positive and gram-negative bacteria at the phylum level after antibiotic treatment.

Figure S3: A rigorous filter of the microbiome data was used to identify taxa of reasonable abundance (> 0.50%) whose frequency may correlate with liver injury, which was quantified using serum ALT levels. (a) Species in Van- or Gen-treated mice. The relative amount of those species in the faecal microbiota was positively or negatively correlated with susceptibility to ConA-induced liver injury. (b) Heatmap of Spearman’s ρ (ρs) correlations between the relative abundance of bacterial species (top 15) in the gut and serum levels of ALT after treatment of mice with ConA. Significant correlations with an FDR-corrected p < 0.05 are indicated with an asterisk (*).

Figure S4: Antibiotic treatment does not change the proportion of liver MDSCs, Treg cells, NKT cells, or IFN-γ-expressing cells. Liver MNCs were isolated from vehicle mice and Van- and Gen-treated mice, and total cells were quantified by FACS (n = 7–8 mice per group). (a) MDSCs (CD11b^+^Gr1^+^), (b) Treg cells (CD4^+^Foxp3^+^), (c) NKT cells (NK1.1^+^TCRb^+^), and IFN-γ-positive cells (NKT cells **(**d, e**)** CD4 cells **(**f**)** SSC **(**g**)** from the mice were detected. Each symbol represents a single mouse. The data are representative of three independent experiments. ns, not significant (t test).

Figure S5: related to Figure 5. Separation strategy and recombination process.

Figure S6: BA-treated mice showed no significant difference in colonic mucosal integrity than the other mice, as observed in colon tissue sections and serum FITC-dextran assay. (a) Images of H&E-stained colonic tissue section obtained at 8 h after ConA administration. (b) FITC-dextran plasmatic fluorescence intensity in the serum of Abx-pretreated mice reconstituted with a single bacterial strain (BA, PD) (n = 8 mice per group).

Figure S7: Vancomycin or gentamycin treatment alters the composition of bile acids in the liver. Metabolome analysis of the liver bile acids in mice treated with antibiotics was performed (n = 6 mice per group). (a) Heatmap of the relative contents of liver bile acids in mice. (b) Bile acids were classified as follows: primary/secondary, unconjugated/conjugated, hydrophilic/hydrophobic. (c) Relative contents of liver bile acids in mice. (d) Taurine-conjugated and glycine-conjugated bile acids. (e) Total bile acids. The bars represent the mean ± s.d. Statistical analyses: t test. **P* < 0.05, ****P* < 0.001.

Figure S8: Metabolome analysis of the liver SCFAs in mice treated with antibiotics was performed (n = 6 mice per group). The bars represent the mean ± s.d. Statistical analyses: t test. **P* < 0.05, ****P* < 0.001.

Figure S9: (a, c, e) Hepa1-6 cells were cocultured with glycine cholic acid (GCA), sodium butyrate (NaB), and sodium propionate (NaP) for 24 h, and CD95-positive cells were quantified by FACS (n = 3 independent biological replicates/group). (b, d, f) Mice were treated with GCA (50 mg kg^-1^ or 100 mg kg^-1^ by gavage), NaB (24 mg kg^-1^ or 80 mg kg^-1^ by gavage), or NaP (50 mM, 100 mM, 200 mM, or 400 mM in the drinking water) for 1 week before ConA administration, and the serum ALT levels were measured at 12 h after ConA administration (n = 8–10 mice per group). The bars represent the mean ± s.d. Statistical analyses: t test.
