# Supplementary material for: Bacteroides acidifaciens in the gut plays a protective role against CD95-mediated liver injury
Source: Gut Microbes. 2022 Feb 6;14(1):2027853. doi: 10.1080/19490976.2022.2027853 (PMC8820816; doi:10.1080/19490976.2022.2027853)
Supplement: Supplemental Material [file KGMI_A_2027853_SM7429.zip › supplementary/Supplementary Information.zip!/Supplementary Information/Supplementary Table 1.docx]

**Table S1.** Related to Figure 5. A list of isolated bacterial strains grown on selective agar media.

| **Strain (Alternative name)** | **Genotype** | **Reference** |
| --- | --- | --- |
| *Bacteroides acidifaciens* | Isolate from murine feces | This study |
| *Parabacteroides distasonis* | Isolate from murine feces | This study |
| *Bacteroides thetaiotaomicron* | Isolate from murine feces | This study |
| *Bacteroides dorei* | Isolate from murine feces | This study |
| *Bacteroides uniformis* | Isolate from murine feces | This study |
