# Supplementary material for: Bacteroides acidifaciens in the gut plays a protective role against CD95-mediated liver injury
Source: Gut Microbes. 2022 Feb 6;14(1):2027853. doi: 10.1080/19490976.2022.2027853 (PMC8820816; doi:10.1080/19490976.2022.2027853)
Supplement: Supplemental Material [file KGMI_A_2027853_SM7429.zip › supplementary/Supplementary Information.zip!/Supplementary Information/Supplementary Table 2.docx]

**Table S2.** Related to Figure 5. A list of bacterial strains isolated from antibiotic-treated mice.

| **Isolation number** | **Mouse genotype** | **16S rRNA-based top hit bacterium**  **> 97% identities** |
| --- | --- | --- |
| **5** | wild-type (Gen) | *Bacteroides dorei* |
| **4** | wild-type (Gen) | *Bacteroides thetaiotaomicron* |
| **3** | wild-type (Gen) | *Bacteroides acidifaciens* |
| **1** | wild-type (Gen) | *Parabacteroides distasonis* |
| **2** | wild-type (Gen) | *Bacteroides uniformis* |
