# Supplementary material for: Bacteroides acidifaciens in the gut plays a protective role against CD95-mediated liver injury
Source: Gut Microbes. 2022 Feb 6;14(1):2027853. doi: 10.1080/19490976.2022.2027853 (PMC8820816; doi:10.1080/19490976.2022.2027853)
Supplement: Supplemental Material [file KGMI_A_2027853_SM7429.zip › supplementary/Supplementary Information.zip!/Supplementary Information/Supplementary Table 3.docx]

**Table S3.** Related to Figure 5. A list of close strains against the strain analyzed by the *Identify* program of NCBI blast.

| **Rank** | **Name** | **Strain** | **Accession** | **Pairwise**  **Similarity(%)** | **Diff/Total**  **nt** |
| --- | --- | --- | --- | --- | --- |
| **5** | *Bacteroides dorei* | *B. dorei 54034* | KP944150.1 | 99.25% | 11/1436 |
| **4** | *Bacteroides thetaiotaomicron* | *B. thetaiotaomicron 1310-29386* | KP944122.1 | 98.20% | 12/1056 |
| **3** | *Bacteroides uniformis* | *B. uniformis W19003B* | KP944145.1 | 99% | 10/1152 |
| **1** | *Bacteroides acidifaciens* | *B. acidifaciens PS4* | LC488237.1 | 97.36% | 18/1136 |
| **2** | *Parabacteroides distasonis* | *P. distasonis* | EU722736.1 | 98.81% | 12/1091 |
