# Supplementary material for: Bacteroides acidifaciens in the gut plays a protective role against CD95-mediated liver injury
Source: Gut Microbes. 2022 Feb 6;14(1):2027853. doi: 10.1080/19490976.2022.2027853 (PMC8820816; doi:10.1080/19490976.2022.2027853)
Supplement: Supplemental Material [file KGMI_A_2027853_SM7429.zip › supplementary/Supplementary Information.zip!/Supplementary Information/Supplementary Table 4.docx]

**Table S4.** Related to Figure S5. Strains and medium used in this study.

| **Strain (Alternative name)** | **Isolate medium (Plate 1)** | **Isolate medium (Plate 2)** |
| --- | --- | --- |
| *Bacteroides acidifaciens* | BHI | BHI + (0.25 g/L) L-Cysteine + (0.25 g/L) Na_2_S 9H_2_O |
| *Parabacteroides distasonis* | YCFA | BHI + (0.25 g/L) L-Cysteine + (0.25 g/L) Na_2_S 9H_2_O |
| *Bacteroides thetaiotaomicron* | BHI | BHI |
| *Bacteroides dorei* | BDS | BHI + (0.25 g/L) L-Cysteine + (0.25 g/L) Na_2_S 9H_2_O |
| *Bacteroides uniformis* | BHI + (0.25 g/L) L-Cysteine + (0.25 g/L) Na_2_S 9H_2_O + (5 mg/ml) Hemin + (10 ug/ml) Vitamin K1 | BHI + (0.25 g/L) L-Cysteine + (0.25 g/L) Na_2_S 9H_2_O + (5 mg/ml) Hemin + (10 ug/ml) Vitamin K1 |
