# Supplementary material for: Bacteroides acidifaciens in the gut plays a protective role against CD95-mediated liver injury
Source: Gut Microbes. 2022 Feb 6;14(1):2027853. doi: 10.1080/19490976.2022.2027853 (PMC8820816; doi:10.1080/19490976.2022.2027853)
Supplement: Supplemental Material [file KGMI_A_2027853_SM7429.zip › supplementary/Supplementary Information.zip!/Supplementary Information/Supplementary Table 5.docx]

**Table S5.** Primers for qpcr in this study.

|  | **Forward** | **Reverse** |
| --- | --- | --- |
| **Fasl** | CAGCTTCAGATGCAAGTGAGTGG | CAAGGACAGAACTCTGACGCTGAC |
| **β-actin** | AGAGGGAAATCGTGCGTGAC | CAATAGTGATGACCTGGCCGT |
| **Fas** | CTCCGAGTTTAAAGCTGAGG | TGTACTCCTTCCCTTCTGTGC |
| **Bcl-2** | AGCCTGAGAGCAACCCAAT | AGCGACGAGAGAAGTCATCC |
| **16s** | AGAGTTTGATCCTGGCTCAG (27F ) | TACGGCTACCTTGTTACGACTT (1492R) |
| **V3–V4 16s** | ACTCCTACGGGAGGCAGCAG (338F) | GGACTACHVGGGTWTCTAAT (806R) |
