# Supplementary material for: Bacteroides acidifaciens in the gut plays a protective role against CD95-mediated liver injury
Source: Gut Microbes. 2022 Feb 6;14(1):2027853. doi: 10.1080/19490976.2022.2027853 (PMC8820816; doi:10.1080/19490976.2022.2027853)
Supplement: Supplemental Material [file KGMI_A_2027853_SM7429.zip › supplementary/Supplementary Information.zip!/Supplementary Information/Supplementary Table 6.docx]

**Table S6.**

| **16S V3-V4 rRNA sequence of representative bacteria of feces DNA isolated from mice consuming antibiotic** | | |
| --- | --- | --- |
| **OTU_28** | *Bacteroides dorei* | TGAGGAATATTGGTCAATGGGCGATGGCCTGAACCAGCCAAGTAGCGTGAAGGATGACTGCCCTATGGGTTGTAAACTTCTTTTATAAAGGAATAAAGTCGGGTATGCATACCCGTTTGCATGTACTTTATGAATAAGGATCGGCTAACTCCGTGCCAGCAGCCGCGGTAATACGGAGGATCCGAGCGTTATCCGGATTTATTGGGTTTAAAGGGAGCGTAGATGGATGTTTAAGTCAGTTGTGAAAGTTTGCGGCTCAACCGTAAAATTGCAGTTGATACTGGATGTCTTGAGTGCAGTTGAGGCAGGCGGAATTCGTGGTGTAGCGGTGAAATGCTTAGATATCACGAAGAACTCCGATTGCGAAGGCAGCCTGCTAAGCTGCAACTGACATTGAGGCTCGAAAGTGTGGGTATCAAACAGG |
| **OTU_204** | *Bacteroides thetaiotaomicron* | TGAGGAATATTGGTCAATGGGCGCAGGCCTGAACCAGCCAAGTAGCGTGAAGGATGACTGCCCTATGGGTTGTAAACTTCTTTTATATGGGAATAAAGTTTTCCACGTGTGGAATTTTGTATGTACCATATGAATAAGGATCGGCTAACTCCGTGCCAGCAGCCGCGGTAATACGGAGGATCCGAGCGTTATCCGGATTTATTGGGTTTAAAGGGAGCGTAGGTGGACAGTTAAGTCAGTTGTGAAAGTTTGCGGCTCAACCGTAAAATTGCAGTTGATACTGGCTGTCTTGAGTACAGTAGAGGTGGGCGGAATTCGTGGTGTAGCGGTGAAATGCTTAGATATCACGAAGAACTCCGATTGCGAAGGCAGCTCACTGGACTGCAACTGACACTGATGCTCGAAAGTGTGGGATATCAACAGG |
| **OTU_11** | *Bacteroides uniformis* | TGAGGAATATTGGTCAATGGACGAGAGTCTGAACCAGCCAAGTAGCGTGAAGGATGACTGCCCTATGGGTTGTAAACTTCTTTTATACGGGAATAAAGTGAGGCACGTGTGCCTTTTTGTATGTACCGTATGAATAAGGATCGGCTAACTCCGTGCCAGCAGCCGCGGTAATACGGAGGATCCGAGCGTTATCCGGATTTATTGGGTTTAAAGGGAGCGTAGGCGGACGCTTAAGTCAGTTGTGAAAGTTTGCGGCTCAACCGTAAAATTGCAGTTGATACTGGGTGTCTTGAGTACAGTAGAGGCAGGCGGAATTCGTGGTGTAGCGGTGAAATGCTTAGATATCACGAAGAACTCCGATTGCGAAGGCAGCCTGCTGGACTGTAACTGACGCTGATGCTCGAAAGTGTGGGTATCAAACAGG |
| **OTU_3** | *Bacteroides acidifaciens* | TGAGGAATATTGGTCAATGGACGAGAGTCTGAACCAGCCAAGTAGCGTGAAGGATGACTGCCCTATGGGTTGTAAACTTCTTTTATATGGGAATAAAATGTTCCACGTGTGGGATTTTGTATGTACCATATGAATAAGGATCGGCTAACTCCGTGCCAGCAGCCGCGGTAATACGGAGGATCCGAGCGTTATCCGGATTTATTGGGTTTAAAGGGAGCGTAGGTGGATTGTTAAGTCAGTTGTGAAAGTTTGCGGCTCAACCGTAAAATTGCAGTTGAAACTGGCAGTCTTGAGTACAGTAGAGGTGGGCGGAATTCGTGGTGTAGCGGTGAAATGCTTAGATATCACGAAGAACTCCGATTGCGAAGGCAGCTCACTGGACTGCAACTGACACTGAGGCTCGAAAGTGTGGGTATCAAACAGG |
| **OTU_44** | *Parabacteroides distasonis* | TGAGGAATATTGGTCAATGGGCGTAAGCCTGAACCAGCCAAGTCGCGTGAGGGATGAAGGTTCTATGGATCGTAAACCTCTTTTATAAGGGAATAAAGTGCGGGACGTGTCCCGTTTTGTATGTACCTTATGAATAAGGATCGGCTAACTCCGTGCCAGCAGCCGCGGTAATACGGAGGATCCGAGCGTTATCCGGATTTATTGGGTTTAAAGGGTGCGTAGGCGGCCTTTTAAGTCAGCGGTGAAAGTCTGTGGCTCAACCATAGAATTGCCGTTGAAACTGGGAGGCTTGAGTATGTTTGAGGCAGGCGGAATGCGTGGTGTAGCGGTGAAATGCTTAGATATCACGCAGAACCCCGATTGCGAAGGCAGCCTGCCAAGCCATGACTGACGCTGATGCACGAAAGCGTGGGGATCAAACAGG |
